# Stereo Online Self-Calibration Through the Combination of Hybrid Cost Functions with Shared Characteristics Considering Cost Uncertainty

**DOI:** 10.3390/s25082565

**Published:** 2025-04-18

**Authors:** Wonju Lee

**Affiliations:** 1 The School of Electrical and Electronic Engineering, Yonsei University, Seoul 03722, Republic of Korea; wonju.lee@yonsei.ac.kr; 2 CTO Division, LG Electronics, Seoul 07796, Republic of Korea

**Keywords:** online calibration, self-calibration, stereo vision

## Abstract

Stereo cameras and stereo matching algorithms are core components for stereo digital image correlation to obtain 3D data robustly in various environments. However, its accuracy heavily relies on extrinsic calibration. In this work, we propose a markerless method for obtaining stereo extrinsic calibration by employing nonlinear optimization on a manifold, which leverages the inherent observability property. To ensure the stability of the optimization and the robustness to outliers when using natural features, we minimize the error constraint between spatial per-frame sparse natural features by stably combining cost functions with similar properties, considering cost uncertainty. Both constraints work in the same direction to reduce the difference in the y-axis coordinates of corresponding points. As a result, the optimization process proceeds smoothly, and it helps reduce the likelihood of overfitting. To extend the problem to the spatiotemporal domain, Bayesian filtering is applied using the logit of zero-shot-based semantic segmentation. Using publicly available data, we conducted experiments where the optimization converged with minimal variation in the number of iterations, and stability was validated through a comparison with state-of-the-art methods.

## 1. Introduction

Stereo cameras and stereo vision have emerged as essential sensing technologies in various applications. Achieving high-quality and deep sensing relies on the precise calibration of the intrinsic parameters of each camera as well as the extrinsic stereo parameters. Temperature changes, shocks, or vibrations not only make calibration necessary in the factory state of the camera but also require periodic zero-point adjustments to maintain the accuracy of the camera measurement device. This is particularly important when conducting detailed experiments, such as stereo digital image correction tests to assess the tension of actual materials or warhead fragmentation analysis experiments, where even the smallest details must be observed.

Efforts to reduce stereo camera calibration errors in existing research can be broadly classified into two categories: offline calibration and online calibration. First, offline calibration includes representative methods such as Zhengyou Zhang’s method [1], which is well suited for narrow-angle cameras and is widely used in image processing (e.g., OpenCV), and Juho Kannala’s method [2], which is more suitable for fisheye cameras. Offline calibration is typically performed for the first time in a factory setting and requires a special chart like a chessboard for direct implementation. Notably, offline calibration generally involves both intrinsic calibration, which corrects lens distortion, and extrinsic calibration, which estimates the camera’s position in 3D space. Among the two major offline calibration methods, Zhang’s approach utilizes a projection model and proposes a closed-form solution using the maximum-likelihood criterion. In contrast, Kannala argues that the perspective projection model is unsuitable for fisheye lenses and suggests a more flexible radially symmetric projection model. In recent trends, Gaku Nakano [3] derived the principal line equation passing through the vanishing point by obtaining the homography between a checkerboard pattern and a camera. Wei Fu [4] combined the strengths of Differential Evolution and Particle Swarm Optimization to enhance global search capabilities and local optimization for chart patterns. Similarly, Yanan Hao [5] used the full factorial analysis method and Latin hypercube sampling to minimize reprojection error and determine the optimal angle and distance for chart patterns. Guohui Wang [6] proposed using the YOLO (You Only Look Once) series to improve checkerboard corner detection. Summarizing these trends, there has been ongoing research in two primary directions: One approach involves using geometric structures such as points or lines extracted from checkerboards to establish and optimize linear or nonlinear equations for camera parameter estimation. The other approach, based on deep learning, focuses on collecting and inferring data to detect checkerboard pattern corners. In terms of application diversity, offline calibration techniques are being extended to multiple cameras [7], underwater cameras [8], and light-field cameras [9]. However, since offline calibration methods rely on additional geometric structures like chessboards, they require knowledge of multiple-view geometry theory. Additionally, the chessboard must be fully visible within the camera’s field of view, and various angles and distances must be measured to ensure stable performance. Due to these constraints, obtaining appropriate image data for offline calibration every time an experiment, such as fragmentation analysis, is conducted to improve accuracy can be challenging. To reduce the inconvenience of repeated calibration, researchers have explored online calibration methods, which have become essential for systems requiring continuous automatic maintenance, such as autonomous vehicles. The key difference between offline and online calibration can be illustrated using robots: if a robot can be calibrated while operating, it is considered online calibration, whereas if the robot’s function must be halted for calibration, it is classified as offline calibration.

A subcategory of online calibration, known as self-calibration, has been proposed to enable calibration without external equipment or special charts like checkerboards. These approaches stem from the inconvenience of traditional offline calibration. Since practical applications require easy access to environmental data, self-calibration research has advanced further than offline calibration, which relies on chessboards. Self-calibration research can be divided into two main approaches. The first estimates both intrinsic and extrinsic parameters simultaneously using geometric information extracted from readily available environments. The second assumes that intrinsic parameters remain nearly unchanged from the factory setting and focuses on estimating extrinsic parameters, which tend to change over time. For intrinsic and extrinsic parameter estimation, Manuel Lopez Antequera [10] used Structure from Motion (SfM) algorithms to predict intrinsic parameters from non-calibrated single images captured in wild environments, training a deep learning model with an emphasis on radial distortion. Chaoning Zhang [11] developed a method specialized for Pan–Tilt–Zoom cameras, where intrinsic parameters change frequently due to zooming. Jiading Fang [12] proposed a self-supervised learning approach to estimate camera parameters from sequential frames. Annika Hagemann [13] introduced a deep learning model using a Bundle Adjustment (BA) layer to estimate camera parameters from consecutive frames and employed a Siamese network for feature extraction using cosine similarity.

However, in practical experiments, camera lenses typically undergo little distortion unless physically damaged, making intrinsic calibration less critical than extrinsic calibration. Consequently, extrinsic calibration has become increasingly necessary due to the cumulative positional shifts that occur during repeated camera use in experiments. For self-calibration methods focused on extrinsic calibration, Eric Dexheimer [14] applied Bayesian sequential estimation based on information theory to update and improve real-time extrinsic parameter correction in multiple camera systems. Deep learning-based approaches include Alexander Tsaregorodtsev’s use of semantic segmentation to extract 2D object boundaries and estimate relative 3D object positions to determine extrinsic parameters [15]. Takayuki Kanai [16] implemented self-supervised learning to generate training data for extrinsic parameter correction. Zhaotong Luo [17] utilized a zero-shot-based Segment Anything Model (SAM) [18] to estimate extrinsic parameters between Lidar and cameras. Jin Gyu Song [19] proposed a CNN-based approach for feature extraction to perform chessboard-free calibration, verified through an attention module. Giacomo D’ Amicantonio [20] estimated homography using deep learning models, while Hongbo Zhao [21] approached stereo calibration from a fundamental perspective based on stereo rectification rather than conventional visual odometry problem solving. Summarizing recent trends, researchers have adopted two strategies for ensuring calibration stability: spatially, they use semantic segmentation techniques to obtain corresponding points based on meaningful geometric structures; temporally, they use camera motion tracking with SfM, BA, or Kalman filtering to increase data diversity across multiple frames, thereby improving calibration robustness. However, this method of estimating the camera pose has a drawback in that it is difficult to apply in experiments such as fragmentation analysis or stereo digital image correlation, where the camera remains fixed during the experiment.

The goal of this paper is to design a feasible algorithm that enables online rectification not only when the camera is moving but also when it is fixed. Therefore, by focusing on the only constraint of stereo rectification, the epipolar constraint, we make the following contributions:

1. We propose a hybrid approach that combines cost functions with similar properties to play complementary roles, ensuring a consistent optimization direction. Specifically, we integrate cost functions with shared characteristics to achieve the *y*-axis alignment of corresponding points and enforce the epipolar constraints of the essential matrix in stereo rectification, considering cost uncertainty. This helps reduce the risk of overfitting and enhances generalization performance.
2. For global optimization for multi-pair cases, particularly to minimize the spatiotemporal noise of corresponding points in stereo cameras, we propose a method that enhances generalized stability through a probabilistic spatial and temporal approach. This involves zero-shot-based semantic segmentation for a spatial probabilistic approach and Bayesian filtering to accumulate relationships between the current and previous frames for a temporal probabilistic approach.
3. The weight of the corresponding points is based not only on the robustness of the feature characteristics but also on the geometry of the camera, using stereo disparity information. This approach is grounded in the fact that, in the perspective view, the area occupied by distant data is significantly smaller than that of nearby data. Therefore, it is proposed that as the pseudo-depth error in stereo geometry increases, the weight of the corresponding points decreases.

## 2. Related Works

This paper proposes a method that can be applied not only when a camera is moving but also when it is fixed. Therefore, related work also considers approaches applicable to both single-pair and multi-pair cases. Hongbo Zhao [21] proposed a method to solve the rectification problem by aligning the *y*-axis. Since the normalized *y*-axes of the left and right cameras must be aligned to ensure that points lie on the epipolar line, the alignment error function can be defined as follows:

(1) $$\frac{r_{l,2}^{⊤}K_{l}^{-1}u_{l,i}}{r_{l,3}^{⊤}K_{l}^{-1}u_{l,i}}-\frac{r_{r,2}^{⊤}K_{r}^{-1}u_{r,i}}{r_{r,3}^{⊤}K_{r}^{-1}u_{r,i}},$$

where *i* is the index of the corresponding point, and $K_{l}$ and $K_{r}$ are the left and right camera parameters in the new coordinate system, respectively. $u_{l}$ and $u_{r}$ are the homogeneous coordinates of the corresponding points in the left and right images, respectively. $r_{l,2}$ and $r_{r,2}$ are the transformation matrices for moving to the new left camera coordinate system and represent the *y*-axis coordinates in the new coordinate system. Similarly, $r_{l,3}$ and $r_{r,3}$ represent the *z*-axis in the new coordinate system. Therefore, this rectification error function is defined as a function that is minimized when the *y*-axis coordinate values, normalized by the *z* values of the corresponding points in the left image and the *y*-axis image, are equal.

Ling and Shen [22] suggested an approach to address the following optimization problem using the epipolar constraint.

(2) $$u_{r}^{⊤}K_{r}^{-T}EK_{l}^{-1}u_{l},$$

where *E* is an essential matrix. However, this method has not been extended to multi-pair cases, and since it uses only a single rotation matrix, the optimization stability may be reduced.

## 3. Methodology

### 3.1. Prerequisites

The rectification of a stereo camera is an extrinsic calibration method that rotates and translates the coordinate systems of the left and right cameras to a common virtual plane, making it possible to search for disparity only in the *x*-axis direction. Ultimately, when there are the rotation matrix *R* and translation vector *t* that align the left camera’s coordinate system with respect to the right camera’s coordinate system, there exist independent rotation matrices for the left and right cameras, $R_{r}={\left[r_{r,1},r_{r,2},r_{r,3}\right]}^{⊤}$ and $R_{l}={\left[r_{l,1},r_{l,2},r_{l,3}\right]}^{⊤}$, respectively. The relationship between them is the following [21]:

(3) $$\begin{array}{c} \begin{array}{cc} R & =R_{r}^{-1}R_{l},\\ t & =-r_{r,1}. \end{array} \end{array}$$

The rotation matrix *R*, which aligns the left camera coordinate system with respect to the right camera coordinate system, must ultimately be equal to the identity matrix and the rotation matrix of the right image plane, $R_{r}$. Therefore, the inverse of $R_{r}$ is applied, and the remaining part becomes $R_{l}$. Additionally, the translation vector *t*, which serves the same purpose, only shifts along the *x*-axis by the baseline distance. Thus, only the first column vector related to the *x*-axis in $R_{r}$ is used, and since the direction is reversed, it takes the negative sign. As a result, the points in the newly rectified coordinate system, or the virtual plane, can be transformed from the left camera coordinate system using the rotation matrix $R_{l}$ and from the right camera coordinate system using the rotation matrix $R_{r}$. These matrices are constructed in stereo rectification to align the coordinate axes in a specific direction.

In stereo rectification, three orthogonal vectors are used to define a new coordinate system. That is, by utilizing a reference vector and the cross product, a new coordinate system is generated and then structured into a rotation matrix. Consequently, the rotation matrix $R_{r}$ is defined by arranging the new coordinate axes as column vectors, and when treating *t* as a unit vector, the original $R_{r}$ can be expressed in the new coordinate system as follows:

(4) $$R_{r}={\left[i_{1},i_{2},i_{3}\right]}^{⊤}.$$

This can be expressed using (3) as follows:

(5) $$R_{r}={\left[\begin{array}{ccc} -t, & i_{3}\times r_{r,1}, & r_{r,1}\times r_{r,2} \end{array}\right]}^{⊤}.$$

The new coordinate axes in stereo rectification are defined as follows: The new *x*-axis, $i_{1}$, is aligned with the direction of the baseline vector *t*, which points from the left camera to the right camera. The new *y*-axis is defined as the cross-product of *t* and the original *x*-axis, ensuring it is perpendicular to both. The new *z*-axis is then defined as the coordinate axis that is orthogonal to both the new *x*-axis and *y*-axis.

### 3.2. Cost Functions with Shared Characteristics

The goal of online self-calibration is to minimize errors in the extrinsic parameters. In this paper, we aim to enhance generalization performance by combining cost functions with similar properties, allowing them to play complementary roles.

The first constraint is designed to ensure the *y*-axis alignment of corresponding points and is defined as follows:

(6) $$\begin{array}{cc} \underset{R_{l},R_{r}}{\mathrm{argmin}}\sum_{i} & {∥\frac{r_{r,2}^{⊤}u_{r,i}^{\prime }}{r_{r,3}^{⊤}u_{r,i}^{\prime }}-\frac{r_{l,2}^{⊤}u_{l,i}^{\prime }}{r_{l,3}^{⊤}u_{l,i}^{\prime }}∥}_{2}^{2},\\ s.t.\; & ∥t∥=1, \end{array}$$

where *i* represents the index of the corresponding point. The matrices $r_{r,2}$ and $r_{l,2}$ are the *y*-axis transformation matrices for the right and left cameras, respectively, used to move to the rectified stereo coordinate system. Similarly, $r_{r,3}$ and $r_{l,3}$ are the *z*-axis transformation matrices for the right and left cameras, respectively, used for the rectified coordinate transformation. Thus, the error is defined based on the normalized coordinate difference between the corresponding points $u_{l}^{\prime }$ and $u_{r}^{\prime }$ in the left and right cameras. The objective of this cost function is to minimize this alignment error. This optimization problem involves adjusting $R_{l}$ and $R_{r}$ to ensure the *y*-axis alignment of the corresponding points. Ideally, if stereo rectification is performed accurately, the corresponding points in the left and right cameras must have the same *y*-coordinate. In other words, the more precise the *y*-axis alignment is, the smaller the error is, and minimizing the error leads to the optimal extrinsic parameter values.

The second constraint is the epipolar constraint based on the essential matrix. This condition states that a point in the left camera must lie on a corresponding line in the right camera. A general 2D line equation can also be expressed in matrix form as follows:

(7) $$\left[\begin{array}{c} a,b,c \end{array}\right]\left[\begin{array}{c} x\\ y\\ 1 \end{array}\right]=0,$$

where *a*, *b*, and *c* are the coefficients that define the line, and *x*, *y*, and 1 represent the coordinates of the point expressed in homogeneous coordinates. If this is converted into the epipolar constraint, it becomes the following:

(8) $$u_{r}^{\prime ⊤}Eu_{l}^{\prime }=0.$$

That is, $u_{r}^{\prime }$ acts as the coefficients of the line (a,b,c), and $Eu_{l}^{\prime }$ acts as the coordinates of the point (x,y,1). Therefore, when a point, $u_{r}^{\prime }$, in the first camera is given, the corresponding point in the second camera must lie on the epipolar line $l^{\prime }=Eu_{l}^{\prime }$. Using the rotation and translation matrices, the epipolar relationship based on the essential matrix is as follows:

(9) $$E={\left[t\right]}_{\times }R,$$

where ${\left|\cdot \right|}_{\times }$ is the skew-symmetric matrix. Using (3), (8), and (9), it can be rewritten as follows:

(10) $$\underset{R_{l},R_{r}}{\mathrm{argmin}}\sum_{i}{∥u_{r,i}^{\prime ⊤}{\left[t\right]}_{\times }R_{r}^{⊤}R_{l}u_{l,i}^{\prime }∥}_{2}^{2}.$$

To summarize the two constraints, the first constraint aims to reduce the difference in the *y*-axis coordinates of corresponding points by gradually adjusting the rotation matrices $\theta_{r}$ and $\theta_{l}$. The second constraint aims to find the rotation and translation matrices based on the condition that when a specific point is marked in one image, the corresponding point must lie on the epipolar line in the other image. By placing the corresponding points on the epipolar line according to the second constraint, the difference in the *y*-axis coordinates of the corresponding points in the left and right images will ultimately be reduced. Therefore, these two constraints consistently have gradients in the same direction. As this conflict is minimized, the optimization process proceeds smoothly, reducing the likelihood of overfitting, much like the probability distribution alignment effect of cross-entropy loss and Kullback–Leibler divergence.

### 3.3. Hybrid Nonlinear Optimization

To iteratively optimize the cost functions (6) and (10), which share similar properties, through the parameterization related to $R_{r}$ and $R_{l}$, the hybrid cost function is defined as follows:

(11) $$\begin{array}{cc} \underset{R_{l},R_{r}}{\mathrm{argmin}}\sum_{i} & {∥\sigma_{1}\left(\frac{r_{r,2}^{⊤}u_{r,i}^{\prime }}{r_{r,3}^{⊤}u_{r,i}^{\prime }}-\frac{r_{l,2}^{⊤}u_{l,i}^{\prime }}{r_{l,3}^{⊤}u_{l,i}^{\prime }}\right)+\sigma_{2}\left(u_{r,i}^{\prime ⊤}Eu_{l,i}^{\prime }\right)∥}_{2}^{2},\\ s.t.\; & ∥t∥=1, \end{array}$$

where $\sigma_{1}$ and $\sigma_{2}$ represent the uncertainty for each cost function. Then, the first-order Taylor expansion of this hybrid cost function is

(12) $$e_{i}\left(R_{l},R_{r}\right)=e_{i}\left(\hat{R_{l}},\hat{R_{r}}\right)+J_{i}\left(\hat{R_{l}},\hat{R_{r}}\right)\cdot \Delta ,$$

where

(13) $$\begin{array}{cc} e_{i} & =\frac{r_{r,2}^{⊤}u_{r,i}^{\prime }}{r_{r,3}^{⊤}u_{r,i}^{\prime }}-\frac{r_{l,2}^{⊤}u_{l,i}^{\prime }}{r_{l,3}^{⊤}u_{l,i}^{\prime }}+u_{r,i}^{\prime ⊤}{\left[t\right]}_{\times }R_{r}^{⊤}R_{l}u_{l,i}^{\prime },\\ \Delta & =\left\{\delta R_{l},\delta R_{r}\right\}\in R^{6}, \end{array}$$

where Δ is the error state vector and J is the Jacobian of $e_{i}\left(R_{l},R_{r}\right)$ with respect to Δ at the current estimate $R_{l}$ and $R_{r}$, as follows:

(14) $$\begin{array}{cc} J_{i}\left(\hat{R_{l}},\hat{R_{r}}\right) & =[\frac{\partial e_{i}\left(\hat{R_{l}},\hat{R_{r}}\right)}{\partial \delta R_{l}},\frac{\partial e_{i}\left(\hat{R_{l}},\hat{R_{r}}\right)}{\partial \delta R_{r}}],\\ \frac{\partial e_{i}\left(\hat{R_{l}},\hat{R_{r}}\right)}{\partial \delta R_{l}} & =\frac{-r_{l,3}^{⊤}u_{l,i}^{\prime }i_{2}^{⊤}{\left[R_{l}u_{l,i}^{\prime }\right]}_{\times }+r_{l,2}^{⊤}u_{l,i}^{\prime }i_{3}^{⊤}{\left[R_{l}u_{l,i}^{\prime }\right]}_{\times }}{{\left(r_{l,3}^{⊤}u_{l,i}^{\prime }\right)}^{2}}\\ \; & +u_{r,i}^{\prime ⊤}{\left[t\right]}_{\times }R_{r}^{⊤}{\left[R_{l}u_{l,i}^{\prime }\right]}_{\times },\\ \frac{\partial e_{i}\left(\hat{R_{l}},\hat{R_{r}}\right)}{\partial \delta R_{r}} & =\frac{r_{r,3}^{⊤}u_{r,i}^{\prime }i_{2}^{⊤}{\left[R_{r}u_{r,i}^{\prime }\right]}_{\times }-r_{r,2}^{⊤}u_{r,i}^{\prime }i_{3}^{⊤}{\left[R_{r}u_{r,i}^{\prime }\right]}_{\times }}{{\left(r_{r,3}^{⊤}u_{r,i}^{\prime }\right)}^{2}}\\ \; & -u_{r,i}^{\prime ⊤}{\left[t\right]}_{\times }{\left[R_{r}^{⊤}R_{l}u_{l,i}^{\prime }\right]}_{\times }. \end{array}$$

To learn the uncertainty $\sigma_{i}$ of the loss functions, the following loss uncertainty regularization is applied [23].

(15) $$E_{i}=\frac{1}{2\sigma_{1}^{2}}e_{1,i}+\frac{1}{2\sigma_{2}^{2}}e_{2,i}+\log\sigma_{1}+\log\sigma_{2},$$

where

(16) $$\begin{array}{cc} e_{i,1} & =\frac{r_{r,2}^{⊤}u_{r,i}^{\prime }}{r_{r,3}^{⊤}u_{r,i}^{\prime }}-\frac{r_{l,2}^{⊤}u_{l,i}^{\prime }}{r_{l,3}^{⊤}u_{l,i}^{\prime }}, \end{array}$$

(17) $$\begin{array}{cc} e_{i,2} & =u_{r,i}^{\prime ⊤}{\left[t\right]}_{\times }R_{r}^{⊤}R_{l}u_{l,i}^{\prime }, \end{array}$$

where $\sigma_{1}$ and $\sigma_{2}$ represent the uncertainties of each cost function, and they are learned to adjust the weights accordingly. Additionally, the $\log\sigma_{i}$ term acts as a regularization to prevent the weights from becoming too small, ensuring that they do not approach zero. By using exp(logσ), the weights are always kept positive. As training progresses, σ gradually converges to the optimal weight, allowing the model to assign lower weights to cost functions with higher uncertainty, as follows:

(18) $$\begin{array}{cc} \delta \sigma_{1} & =\frac{e_{i,1}}{\exp{\left(\log\sigma_{1}\right)}^{2}}-\frac{1}{\exp\left(\log\sigma_{1}\right)}, \end{array}$$

(19) $$\begin{array}{cc} \delta \sigma_{2} & =\frac{e_{i,2}}{\exp{\left(\log\sigma_{2}\right)}^{2}}-\frac{1}{\exp\left(\log\sigma_{2}\right)}. \end{array}$$

### 3.4. Spatiotemporal Filtering for Multi-Pair Cases

The extrinsic calibration ultimately depends on the quality of the corresponding points between the left and right cameras. Therefore, it is essential to extract corresponding points that are resilient to sensor noise and outliers, which is why a robust feature extractor can meaningfully capture characteristics from multiple camera frames. Among deep learning models, those designed to generalize based on a large amount of general environmental data are called foundation models, and this paper aims to incorporate such models. One such model, the SAM [18], is a powerful semantic segmentation model that distinguishes boundaries between backgrounds and foregrounds. It can divide spatially meaningful semantic areas in an image and generate masks for various objects. In other words, the SAM outputs a mask that includes the probability (in logit form) of each pixel belonging to a specific object. The logit values are typically positive for high object presence probability and negative for low object presence probability. However, since the SAM’s output corresponds to information for a single frame, post-processing is required to maintain consistent region information across consecutive frames. To address this, Bayesian filtering can be applied to accumulate the SAM’s logit output over time. The reason for using the logit is that it provides continuous confidence values, making it easier to accumulate information across frames by applying Bayesian filtering. In other words, the confidence can be gradually adjusted over time to eliminate noise and allow for stable tracking. Bayesian filtering is a method of updating the probability distribution over time, combining information from previous frames and observations from the current frame to make more accurate estimates.

The initial state for the prior is set to ensure the maintenance of pixel-wise probability distributions, where the object presence probability for each pixel is initially set as follows:

(20) $$P\left(O_{k}|L_{k}\right)=\tau \left(L_{k}\right),$$

where $O_{k}$ is the probability of an object existing at the time *k*, $L_{k}$ is the logit output of the SAM, and τ is the sigmoid function that converts the logit into a probability.

In stereo vision, the depth *Z* is calculated as follows using the disparity *d* between two images captured by two cameras with a baseline:

(21) $$Z=\frac{fb}{d},$$

where *f* is a focal length and *b* is a baseline. And, if both sides are differentiated with respect to *d*,

(22) $$\Delta \hat{Z}\approx -\frac{fb}{\hat{d^{2}}}\cdot \Delta d.$$

where $\hat{d}$ is a pseudo-disparity by computing the disparity before calibration. Therefore, the relative depth error is the following:

(23) $$\frac{\Delta \hat{Z}}{Z}\approx \frac{\Delta d}{\hat{d}}.$$

For closer objects, the disparity is larger, while for distant objects, the disparity $\hat{d}$ is smaller. In other words, as the depth increases, the value of the disparity $\hat{d}$ decreases, but the disparity detection error Δd does not change. Therefore, as the depth increases, the relative depth error $\frac{\Delta \hat{Z}}{Z}$ becomes larger. In other words, as the disparity $\hat{d}$ decreases, small errors lead to larger depth errors. We define the pseudo-depth $\Delta \hat{Z}$ based on depth error properties to compute the disparity between corresponding points before calibration. Hence, using (20) and (22), we define the prior probability as follows:

(24) $$P\left(O_{k}|L_{k}\right)=\tau \left(L_{k}\right)\eta \;\frac{1}{\Delta \hat{Z}},$$

where η is a normalization.

In the initial frame, the logit values of the SAM are converted into probabilities to set the initial distribution. Then, in the prediction step, Bayesian filtering uses the probability distribution from the previous time step k−1 to generate the prediction for the current time step *k*. We apply the Markov Assumption at the pixel level:

(25) $$P\left(O_{k}|L_{1:k-1}\right)=\int P\left(O_{k}|O_{k-1}\right)P\left(O_{k-1}|L_{1:k-1}\right)\;dO_{k-1},$$

where $P(O_{k}|O_{k-1})$ is the state transition probability where the pixel state remains, and $P(O_{k-1}|L_{1:k-1})$ is the probability distribution up to the previous frame. Finally, the update step reflects the new logit output of the current SAM to update the probabilities. We use Bayes’ theorem:

(26) $$P\left(O_{k}|L_{1:k}\right)=\frac{P\left(L_{k}|O_{k}\right)P\left(O_{k}|L_{1:k-1}\right)}{P(L_{k}|L_{1:k-1})}.$$

Revisiting the basic concept of Bayesian filtering, it is typical to calculate the posterior by multiplying the prior and the current observation likelihood. This is because using probability addition can cause probabilities to exceed 1 and temporal continuity is not guaranteed, while using probability multiplication ensures that probabilities remain within the range of 0 to 1, and low-confidence values are automatically reduced. As a result, the updated cumulative probability map gradually forms a more accurate object mask over time. In other words, in the presence of noise, Bayesian filtering forms a smooth object probability and generates a reliable mask. Pixels with initially low probabilities also improve the object mask as the probabilities accumulate over multiple frames.

## 4. Experimental Results

### 4.1. Theoretical Feasibility

We used an Intel i7-9750H CPU @ 2.60GHz, with 32.0GB of RAM, and an Nvidia GeForce RTX 2080 with Max-Q Design 8GB GPU for all experiments. Our software was developed using Python 3.8 and built with the Scipy, Kornia, and OpenCV libraries. It was based on the publicly available online self-calibration source code by Hongbo Zhao [21]. The proposed implementation is summarized in Algorithm 1.
**Algorithm 1** Hybrid nonlinear optimization.**Require:** 
$K_{r},K_{l}$, ||t||  1:$\sigma_{1}=1,\;\sigma_{2}=1$  2:$R_{l}={\left[0,0,0\right]}^{⊤},\;R_{r}={\left[0,0,0\right]}^{⊤}$  3:$c=1e^{-6},\;lr=1e^{-6}$,  4:$P\left(O_{0}|L_{0}\right)=\tau \left(L_{0}\right)$  5:**for** *k* frames **do**  6:    $P(O_{k}|L_{1:k})=$ (26)  7:    **while** $|E_{j-1}^{2}-E_{j}^{2}|\;\leq \;c$ **do**  8:        **for** i≤N **do**  9:           $\Delta \hat{Z}\leftarrow$ (22)10:           Extract corresponding points $u_{r,i},u_{l,i}$11:           $u_{r,i}^{\prime }=K_{r}^{-1}u_{r,i}$12:           $u_{l,i}^{\prime }=K_{l}^{-1}u_{l,i}$13:           $e_{i,1}=$ (16)14:           $e_{i,2}=$ (17)15:           $E_{i}=$ (15)16:           $\delta \sigma_{1}=$ (18)17:           $\delta \sigma_{2}=$ (19)18:           $\log\sigma_{1}\leftarrow lr\cdot \delta \sigma_{1}$19:           $\log\sigma_{2}\leftarrow lr\cdot \delta \sigma_{2}$20:        **end for**21:        $\left\{\delta R_{l,j},\delta R_{r,j}\right\}=-{\left(J^{⊤}J+\lambda I\right)}^{-1}J^{⊤}E$22:        ${\hat{R}}_{l,j}\leftarrow {\hat{R}}_{l,j-1}\delta R_{l,j-1}$23:        ${\hat{R}}_{r,j}\leftarrow {\hat{R}}_{r,j-1}\delta R_{r,j-1}$24:    **end while**25:    $P\left(O_{k-1}|L_{1:k-1}\right)=P\left(O_{k}|L_{1:k}\right)$26:**end for**27:$R^{*}={\hat{R}}_{r}^{⊤}{\hat{R}}_{l}$28:$t^{*}=\left||t|\right|\cdot {\hat{r}}_{r,1}$

The experiments were conducted using publicly available crack fragmentation simulation data and real data from Duo Digital Image Correlation (DIC) [24], a publicly available stereo DIC dataset for stress–strain analysis. The results were compared with the latest study by Hongbo Zhao and proceeded as follows:

1. We fixed the right camera as a reference and conducted quantitative performance tests by adjusting the left camera at different angles while increasing the outlier ratio.
2. The number of optimization iterations was quantitatively analyzed compared to Hongbo Zhao’s algorithm, and the appropriateness of cost reduction was verified.
3. To evaluate the performance of spatiotemporal filtering, qualitative measurements of temporal variations were conducted using fragmentation simulations.
4. Using stress–strain analysis data, a qualitative evaluation of disparity estimation was performed in comparison with Hongbo Zhao’s algorithm.

#### 4.1.1. Quantitative Evaluation of Extrinsic Parameter Estimation

In the quantitative evaluation of extrinsic parameter estimation, particularly on the outdoor KITTI2015 dataset [25,26], when the outlier ratio was set between 0% and 20%, the results of the paired t-test showed no statistically significant difference. However, when the outlier ratio was increased to 30–50% of the entire dataset, the paired *t*-test indicated that the two datasets were statistically different. The one-tailed *p*-value was $9.65\times 10^{-12}$ and the two-tailed *p*-value was $1.93\times 10^{-11}$, both of which were close to zero. This strongly supports the rejection of the null hypothesis. In other words, there was a statistically significant difference between the two groups, with both the one-tailed and two-tailed tests confirming this significance. This result provides strong evidence that the two groups were indeed different.

As shown in Table 1, the performance improvement of the proposed method became evident as noise increased on the outdoor KITTI2015 dataset. On the clean dataset, no statistically significant difference was observed, meaning the difference was not substantial. Additionally, the results of the *F*-test indicated that the variability in the data in the two experimental groups was similar. Specifically, the *F*-statistic was 1.0066, which was very close to 1, suggesting that the variances of the two groups were nearly identical. The right-tailed *p*-value was 0.4850, and the two-tailed *p*-value was 0.970, both greater than 0.05. Thus, the null hypothesis could not be rejected, meaning there was no evidence that the variances of the two groups were different. Consequently, the assumption of equal variance was valid for the *t*-test. It implied the high reliability of the data and consistency in the experimental conditions. It also suggested that any observed mean difference was likely due to a real effect rather than random variation, allowing for valid statistical analysis without adjustments.

#### 4.1.2. Convergence Speed Evaluation

For evaluating the optimization convergence speed, the number of additional training iterations for loss uncertainty was excluded from this experiment, and the ratio of the two cost functions was set to 5:5. The number of iterations required for convergence was determined by checking when the cost function change became smaller than a certain threshold. In the indoor scenario, both the one-tailed and two-tailed *t*-tests resulted in *p*-values greater than 0.05, meaning that the null hypothesis could not be rejected. This indicates that the data did not show a statistically significant difference or effect.

- One-tailed *p*-value: 0.2722 → This meant that under the null hypothesis, the probability of obtaining the observed value or a more extreme one was 27.22%. Since this value was greater than 0.05, there was insufficient evidence to reject the null hypothesis.
- Two-tailed *p*-value: 0.5445 → This meant that the probability of obtaining an equally extreme value in either direction was 54.45%, which was much higher than 0.05. This further confirmed that the result was not statistically significant.

Conversely, in the outdoor scenario, both the one-tailed and two-tailed *t*-tests resulted in *p*-values smaller than 0.05, meaning that the null hypothesis was rejected, and the alternative hypothesis was accepted. This indicated a statistically significant difference, suggesting that the data did not support the null hypothesis.

- One-tailed *p*-value: 0.0031 → This meant that the probability of obtaining a more extreme value under the null hypothesis was 0.31%, which was much smaller than 0.05. Thus, the result was statistically significant, providing strong evidence to reject the null hypothesis and accept the alternative hypothesis.
- Two-tailed *p*-value: 0.0061 → This meant that the probability of obtaining an equally extreme value in either direction was 0.61%, which was also much smaller than 0.05. Again, this confirmed statistical significance and supported rejecting the null hypothesis.

As shown in Table 2, the convergence speed in the outdoor scenario was found to differ from the baseline. However, the difference was marginal, typically within one to two iterations.

#### 4.1.3. Qualitative Experimental Results

**Figure 1:**
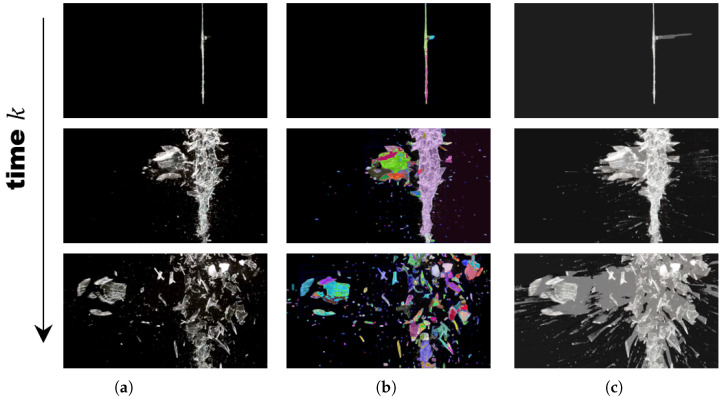
The qualitative experimental results of spatiotemporal filtering during fragmentation simulations. The vertical axis represents frames over consecutive time steps. (**a**) Raw consecutive images. A simulation of glass breaking due to a bullet impact. (**b**) Spatial filtering only, without temporal Bayesian filtering. Colors indicate different regions. (**c**) Our results. It is possible to observe the trace of the crack’s movement.

**Figure 2:**
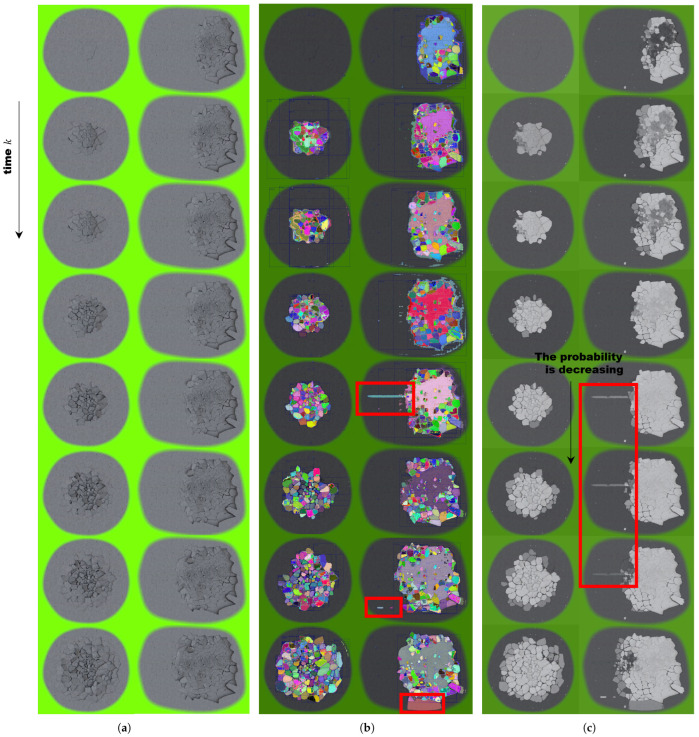
The qualitative experimental results of spatiotemporal filtering during crack fragmentation simulations. The vertical axis represents frames over consecutive time steps. (**a**) Raw consecutive images. Experiments were conducted on two separate simulations. (**b**) Spatial filtering only, without temporal Bayesian filtering. The red boxes indicate the SAM’s misrecognition [18]. Colors indicate different regions. (**c**) Spatiotemporal filtering. The higher the probability was, the whiter the color displayed is. The probability of misrecognition gradually decreased over time.

We experimented with spatiotemporal filtering to reject misrecognitions, as any deep learning method utilizing zero-shot semantic segmentation inevitably includes misrecognitions, as shown in Figure 1 and Figure 2. Bayesian filtering, though a simple method for handling new data in the presence of existing data, yields strong results. The only caveat is that the update speed needs to be properly adjusted, and if the minimum and maximum values of the updated probabilities are not constrained, the system will fail to update even with new incoming data. This filtering technique increases the probability over time, allowing the trace of the past path to be observed.

**Figure 3:**
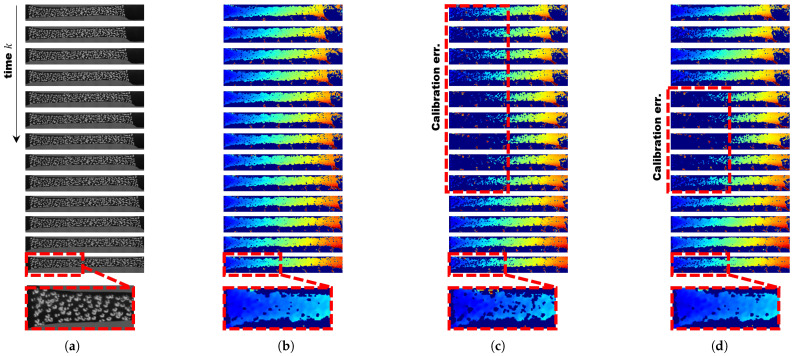
A qualitative comparison of disparity estimation using stress–strain analysis data using SGM [29] via OpenCV as the traditional disparity estimation method. Every time, a 10% noise ratio and 2-degree rotation were applied. The Jet color map has been used to represent the disparity map, with larger values shown in red and orange, and smaller values shown in blue. (**a**) Raw sequential right camera images captured during stress–strain experiments. (**b**) Estimated disparity maps using rectified stereo images using traditional extrinsic calibration or offline calibration [1]. (**c**) Disparity maps estimated using stereo images rectified based on the extrinsic parameters estimated using Hongbo Zhao’s algorithm [21]. (**d**) Disparity maps estimated using online self-calibration based on the extrinsic parameters estimated using our proposed algorithm.

We conducted experiments using publicly available DuoDIC data [24], as shown in Figure 3, to analyze the impact of online self-calibration on disparity estimation based on stress–strain analysis data. Figure 3b illustrates the results using traditional offline calibration, where no noise or image tilt was applied. Since most deep learning-based disparity estimation methods are trained on datasets such as ETH3D [27], Middlebury [28], and KITTI, they often misinterpret the dot pattern sprayed on the test material as an object for recognition, resulting in inaccurate disparity estimation. Therefore, we employed Semi-Global Matching (SGM) via OpenCV [29] as the traditional disparity estimation method for our experiment. Unlike deep learning-based methods, SGM lacks robustness to even minor misalignment. Thus, we tested it with a small 2-degree tilt and 10% noise. Meanwhile, the algorithm proposed by Hongbo Zhao exhibited a tendency for accumulated rotation bias in one direction, as shown in Table 1, in the indoor scenario, though this effect was not distinctly visible on the disparity map.

**Figure 4:**
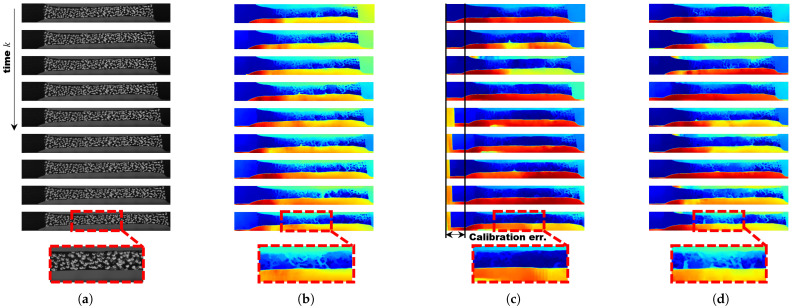
Qualitative comparison of disparity estimation using stress–strain analysis data. The deep learning method RAFT-Stereo by Lahav and Lipson [30] was used for disparity estimation. Every time, a 10–50% noise ratio and 2 deg.–10 deg. rotation were randomly applied. In the disparity map, the focus is more on how clearly the crack is visible rather than the meaning of the colors. (**a**) Raw sequential right camera images captured during stress–strain experiments. (**b**) Estimated disparity maps using rectified stereo images using traditional extrinsic calibration or offline calibration [1]. (**c**) Disparity maps estimated using stereo images rectified based on the extrinsic parameters estimated using Hongbo Zhao’s algorithm [21]. (**d**) Disparity maps estimated using online self-calibration based on the extrinsic parameters estimated using our proposed algorithm.

For deep learning-based disparity estimation, we used RAFT-Stereo by Lahav and Lipson [30] as shown in Figure 4. While the method naturally aligned the vertical axis, it failed to properly align the horizontal axis, directly reflecting the calibration error. Since the model recognized the dot pattern itself as the target for distance measurement, the results differed from traditional methods. However, from a positive perspective, this approach provided insight into the crack severity of the experimental material, suggesting that such a method could have meaningful applications. Nevertheless, using a model trained on an active stereo camera dataset would likely yield more reliable results.

### 4.2. Deployment Considerations

This section aims to evaluate the practical feasibility of applying the proposed method in practice. The practical applicability largely depends on the extent to which the additional computational load—introduced by the proposed algorithm on top of the existing system load, including communication overhead—can be optimized. To mitigate this issue, the proposed experiment employed algorithmic complexity reduction strategies, such as problem simplification and initial value estimation. Furthermore, the feasibility of the proposed approach was validated using a recently published stereo vision benchmark, FoundationStereo [31].

#### 4.2.1. Problem Simplification for Low Complexity

**Figure 5:**
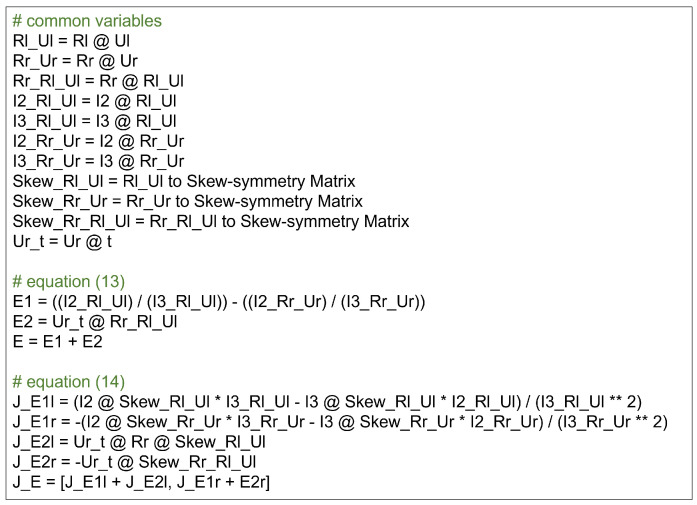
Eliminating redundant calculations and caching for cost functions. The symbol * represents multiplication, and ** represents exponentiation (squared).

The first obvious attempt to improve optimization was simplifying the problem. In particular, eliminating redundant computations and applying caching were among the optimization techniques used to avoid repeated calculations by storing and reusing results when needed. To prevent the repeated execution of identical operations, previously computed results were stored in variables or memory for reuse. Although caching increases memory usage, it is efficient if the reduction in computation time outweighs the cost. This also reduces complexity, results in cleaner code structure, and eliminates unnecessary computations. As shown in Figure 5, there are parts within the cost functions (13) and (14) where identical terms are repeatedly used. By caching these, we aimed to reduce computation and speed up the process. The number of Multiply–Add operations changed using caching was reduced as follows:

- Number of addition operators (+): 11→5 (vs. Hongbo Zhao: 2).
- Number of subtraction operators (−): 37→37 (vs. Hongbo Zhao: 22).
- Number of multiplication operators (∗): 63→58 (vs. Hongbo Zhao: 52).
- Number of division operators (/): 11→7 (vs. Hongbo Zhao: 7).
- Number of matrix multiplication operators (@): 43→25 (vs. Hongbo Zhao: 31).

Although caching was employed, the method lacked sufficient real-time capability to be considered an online approach. Therefore, to support continuous systems, it was necessary to introduce a technique that could fundamentally accelerate the convergence of the nonlinear optimization process.

#### 4.2.2. Covariance-Based Search Space Reduction for Initial Guesses

Next, we aimed to enhance the applicability of the proposed methodology in continuously operating systems by accelerating the optimization process through narrowing the solution space of the nonlinear equations we aimed to solve. In other words, we sought to gradually reduce the search space over time by estimating better initial guesses and shrinking the confidence bounds when the solution changes were small, much like how a Kalman filter dynamically adjusts prediction uncertainty. This approach, which dynamically adjusted the confidence interval, was expected to improve convergence and significantly reduce computational load.

In particular, to improve the initial guess, we designed a mechanism that dynamically constrained the solution space using the parameter covariance derived from the Jacobian of the error of the previous frame’s optimization result. During repeated optimization, the uncertainty from the parameter covariance was evaluated: if uncertainty decreased, the search space was tightened; if uncertainty increased, the confidence region was widened accordingly. In this way, we implemented a strategy that modeled the time-varying uncertainty of the solution and progressively adjusted the optimization range. The use of a confidence interval was based on the assumption that “the optimized parameters are highly likely to lie within this range”.

To summarize the proposed mechanism, we predicted the high-probability solution region based on the parameter covariance matrix calculated during optimization and automatically adjusted the search range through the following steps:

1. Compute the Jacobian of the error from the previous frame’s optimization result.
2. Approximate the parameter covariance as the Jacobian of the error to define the confidence interval.
3. Define rules for the dynamic adjustment of the range for the current initial value.
4. Iteratively reduce the range using dynamic updates.

These rules were applied repeatedly until convergence. The range was narrowed when the covariance decreased and widened when the covariance increased, enabling the real-time adjustment of the search space.

In terms of computational cost, calculating the parameter covariance is not particularly complex, as it is approximated in a way that allows the reuse of the Jacobian of the error (14) already computed by the optimization algorithm, Levenberg–Marquardt (LM). Additionally, the scaling factor of the covariance—the variance of the error—is also typically derived from already computed values (15). First, we approximated the covariance of the rotation and translation parameters using the Jacobian of the error, as follows:

(27) $$\sum_{\hat{R}}\approx J_{E}^{⊤}\sum_{E}J_{E},$$

where $\sum_{\hat{R}}$ is the covariance of the estimated parameters we aim to obtain. $\sum_{E}$ is the error covariance matrix. $J_{E}$ is the Jacobian of the error term. This Jacobian is a sensitivity matrix that represents how the error is influenced by the parameters, and it plays the role of transforming the error covariance into the parameter space. Also, assuming isotropic Gaussian noise with $\sum_{E}=\sigma^{2}I$, the parameter covariance to be estimated can be rewritten as follows:

(28) $$\sum_{\hat{R}}\approx \sigma^{2}{\left(J_{E}^{⊤}J_{E}\right)}^{-1},$$

where

(29) $$\sigma^{2}=\frac{\sum {\left(E_{i}\right)}^{2}}{N-p},$$

where *N* is the number of data points, and *p* is the number of model parameters. $J^{T}J$ is already computed by LM in Line 21 of Algorithm 1, and the denominator of the error is already computed in the error expression (15), so the only additional calculation required is dividing by N−6 (the number of parameters). If this adjustment for degrees of freedom (Bessel’s Correction) is not made, the model may become biased as it tends to fit the data perfectly. Therefore, to reduce the degrees of freedom by the number of model parameters, we divide by N−p. In other words, since the optimization algorithm adjusts the parameters to minimize the error, it is designed to divide by this value to avoid biased variance estimation, considering the degrees of freedom. The entire algorithm is summarized in Algorithm 2.
**Algorithm 2** Covariance-based search space reduction for initial guesses.**Require:** 
$R_{k}^{*},\;N,\;\sum {\left(E_{i}\right)}^{2},\;{\left(J_{E}^{⊤}J_{E}+\lambda I\right)}^{-1}$  1:p=6  2:α=0.5,β=2.0  3:threshold=0.015  4:confidence_range=0.001  5:$\sum_{\hat{R}}\leftarrow$ (28)  6:$uncertainty=\sqrt{diag(\sum_{\hat{R}})}$  7:**if** mean(uncertainty)≤threshold **then**  8:    confidence_range=confidence_range∗α  9:**else**10:    confidence_range=confidence_range∗β11:**end if**12:$R_{k+1}^{0}=R_{k}^{*}+random\left(-confidence\_range,confidence\_range\right)$

Considering the rapid advancements in the field, we utilized the most recent benchmark dataset, FoundationStereo [31], specifically using the manipulation-v5-realistic-kitchen-2500-1 (indoor) and amr-v5-b6-realistic-outdoor-intersection-vehicled-1000-7 (outdoor) sections for our experiments. In this dataset, to ensure broad applicability across various stereo cameras, the stereo baseline was randomly sampled from a Normal (0.15, 0.02) or Uniform (0.05,0.20) distribution, while the focal length was determined based on Normal (2.87343,0.25) or Uniform (1.5,6.75) distributions. Therefore, in our experiments, we fixed the stereo baseline at 0.15 and assumed a sensor size of 5 mm, resulting in a focal length of 735 pixels. Most importantly, in order to evaluate the applicability of the proposed method in a continuous system, we treated the dataset as a sequential stream. Every five frames, we randomly rotated the yaw angle of the left camera in the stereo pair between 1 to 10 degrees, while keeping the roll and pitch angles fixed at 0 degrees. In the image plane, yaw rotation affected both the *x*-axis and the *y*-axis simultaneously.

**Figure 6:**
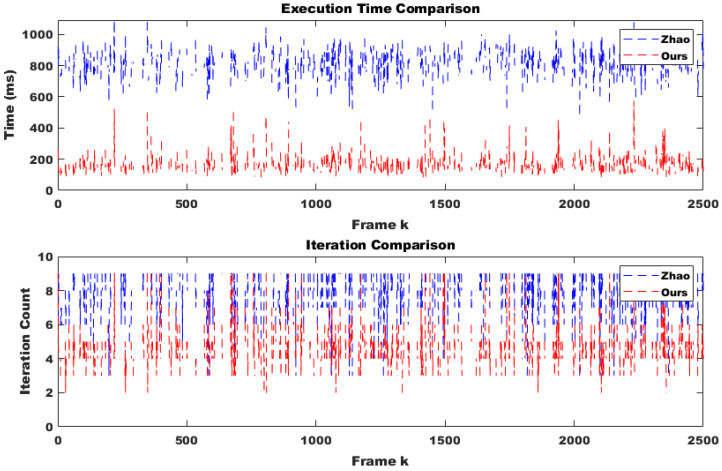
Comparison of the execution time and optimization iteration produced by Hongbo Zhao’s model [21] and ours under identical condition during sequential indoor frames. The rapid increase in the computational time of the proposed method was predominantly observed in scenarios where frame continuity was disrupted and a significant discrepancy existed between the GT of the previous and current frames.

**Figure 7:**
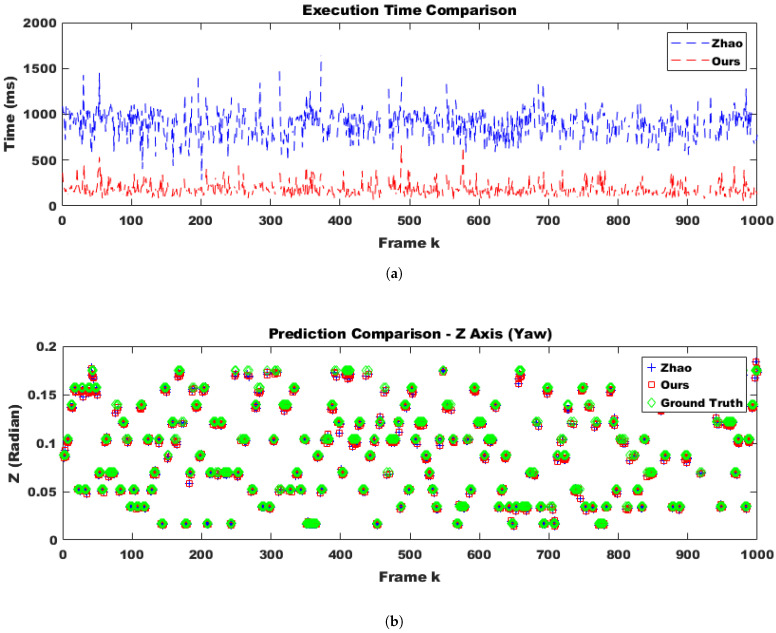
Comparison using an outdoor dataset: execution time and *Z*-axis prediction produced by Hongbo Zhao’s model [21] and ours under identical condition. (**a**) The execution time was longer in outdoor environments, which was due to the larger number of highly reliable corresponding points. Therefore, it seemed desirable to exclude more than 1000 points from the computation. (**b**) A comparison of *Z*-axis prediction. Compared to indoor environments, the prediction accuracy was higher due to the presence of highly reliable corresponding points. Every 5 frames, the yaw angle of the left camera in the stereo pair was randomly rotated between 1 and 10 degrees, while the roll and pitch angles were fixed at 0 degrees.

**Figure 8:**
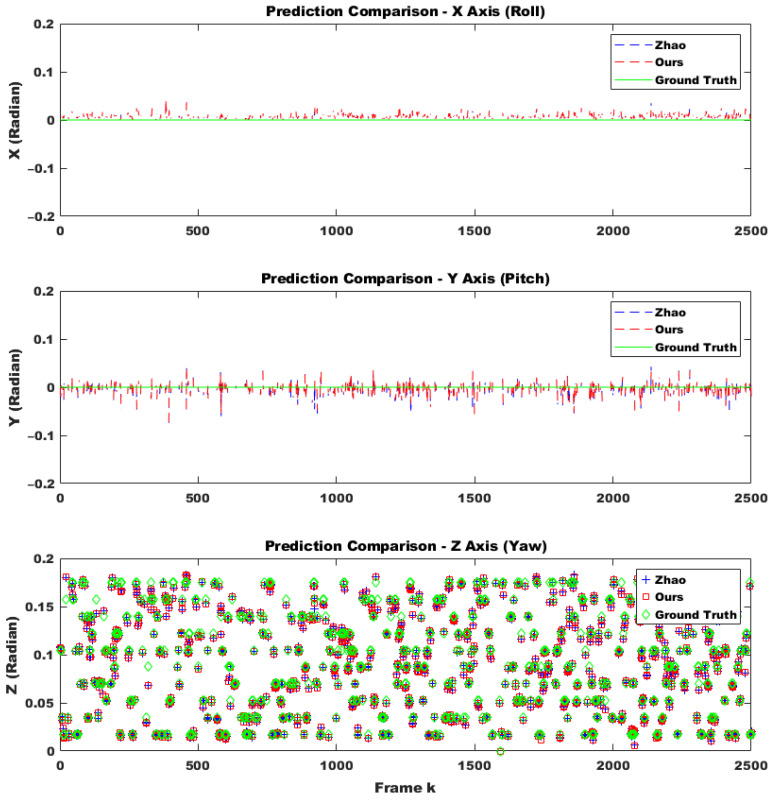
Comparison of the prediction produced by Hongbo Zhao’s model [21] and ours under identical condition regarding 3 axes using covariance-based search space reduction for initial guesses during sequential indoor frames. Every 5 frames, the yaw angle of the left camera in the stereo pair between 1 to 10 degrees was randomly rotated, while the roll and pitch angles were kept fixed at 0 degrees.

**Figure 9:**
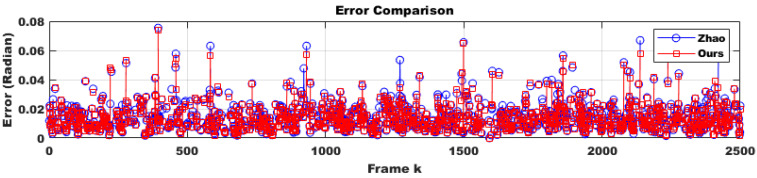
Comparison of the errors produced by Hongbo Zhao’s model [21] and ours under identical condition during sequential indoor frames. Error = $||GT-R^{*}{\left|\right|}_{2}$. The results indicate that the accuracy of the two methods did not differ significantly.

**Figure 10:**
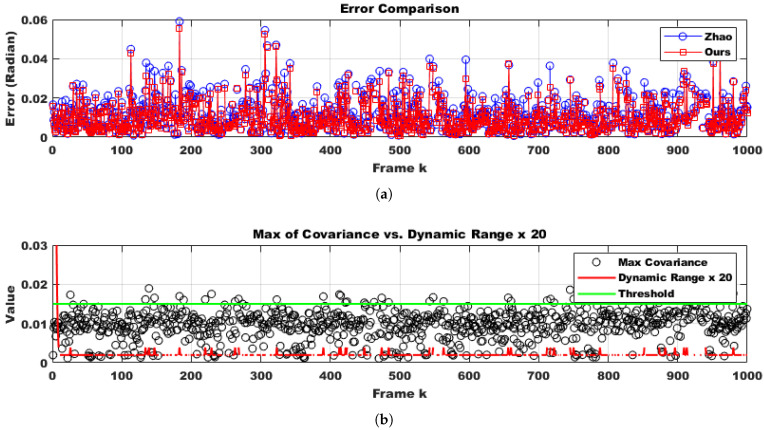
Error comparison produced by Hongbo Zhao’s model [21] and ours using an outdoor dataset and dynamic confidence interval analysis using an outdoor dataset. (**a**) Compared to the indoor environment, the absolute value of the error decreased due to the presence of more reliable corresponding points. (**b**) The dynamic confidence interval also showed less variation compared to the indoor case.

**Figure 11:**
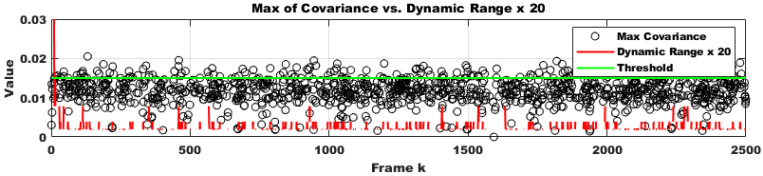
Covariance-based search space reduction for initial guesses increased the trusted dynamic range when the maximum of the covariance exceeded a threshold and conversely reduced the dynamic range when it fell below the threshold, thereby gradually improving convergence of the solution during sequential indoor frames.

To evaluate the robustness of the proposed method in a practical system, we selected only difficult samples with a matching confidence lower than 0.25 as corresponding points. Furthermore, 30% of these points were replaced with outliers. From the perspective of deploying the system in real-world scenarios, performing calibration in every frame would impose a significant load on the system and should be avoided. Additionally, extreme impacts that might physically damage the stereo camera are outside the scope of online calibration. Therefore, it is appropriate to use the online calibration algorithm only at moments when the conditions suggest a high likelihood of successful calibration, which also minimizes overall system load. Through continuous testing, we empirically found that the proposed method could successfully compute calibration results only when the number of corresponding points exceeded 500 and at least two optimization iterations were performed. Moreover, since we randomly altered the camera’s tilt every five frames, excluding frames where calibration was unlikely to succeed and could disrupt temporal continuity, making it more difficult to adjust the initial parameter confidence interval using covariance. Despite these adverse conditions, as shown in Figure 6 (indoor) and Figure 7a (outdoor), the proposed method was approximately four times faster in execution compared to the approach by Hongbo Zhao. This improvement was due to a reduction in the number of iterations by about half, as well as the conversion of Python code to C using the Eigen open-source library. Furthermore, even with this faster convergence, the accuracy across all three rotational axes was comparable to that of Hongbo Zhao, as illustrated in Figure 7b (outdoor) and Figure 8 (indoor). The L2 norm error between our results and the ground truth (GT), shown in Figure 9 (indoor) and Figure 10a (outdoor), also demonstrated similar performance. This is attributed to the proper adjustment of the initial parameter confidence interval through the maximum and threshold of the covariance in each frame, as visualized in Figure 10b (outdoor) and Figure 11 (indoor). The decrease in the diagonal elements of the covariance matrix indicated an increase in the reliability of the initial estimate. In other words, a smaller covariance implied a better model fit to the data, while a larger covariance resulted in a wider confidence interval, signaling higher model uncertainty. As can be seen from the differences in the experimental results between the indoor and outdoor datasets, the covariance played a crucial role in reflecting the uncertainty of the model and in evaluating both the confidence intervals and the accuracy of parameter estimation, which was validated through our experiments. Ultimately, by gradually narrowing the solution space using the covariance of rotation and translation parameters, our method achieved a 1.5-times improvement in optimization speed over the Hongbo Zhao method, while maintaining comparable accuracy in solving the stereo camera extrinsic calibration problem.

## 5. Conclusions

This study proposes a method for stably combining cost functions with similar approaches for online self-calibration. In stereo rectification, constraints are imposed only on the *y*-axis alignment of corresponding points, without restrictions on the *x*-axis or *z*-axis. Therefore, this study also focused on *y*-axis alignment and made efforts to integrate constraints with similar properties. To fulfill the objectives of an online method, the algorithm was progressively accelerated by the means of approximate initial value estimation, with the goal of improving practical feasibility in continuous system operation. The proposed method was quantitatively validated through paired *t*-tests on publicly available indoor and outdoor datasets and was qualitatively evaluated by linking it to disparity estimation, demonstrating its potential.

As a direction for future research, one limitation of this study is its focus on the rotation matrix, leaving the treatment of the translation matrix relatively insufficient. To enhance stability, further integrating an explicit constraint on the translation matrix is suggested. Finally, deep learning-based approaches for online self-calibration are discouraged due to the resource limitations of embedded systems. Instead, we argue that addressing the problem as a geometric nonlinear optimization is the more appropriate approach.

## Figures and Tables

**Table 1:** A quantitative comparison of the extrinsic parameter estimation $||GT-R^{*}{\left|\right|}_{2}$ between our proposed algorithm and [21] on indoor (DuoDIC [24]) and outdoor (KITTI2015 [25,26]) datasets by adjusting the left camera at different angles while increasing the outlier ratio.

Scenario	Outlier Ratio	Algorithm	0 deg.	1 deg.	2 deg.	3 deg.	4 deg.	5 deg.	6 deg.	7 deg.	8 deg.	9 deg.	Mean ↓	Std. ↓
Indoor	0%	[21]	0.1532	0.0722	0.1596	0.1105	0.1282	0.1421	0.1356	0.1222	0.1369	0.1531	0.1314	0.0257
		Ours	0.0373	0.0251	0.0400	0.0301	0.0339	0.0409	0.0377	0.0351	0.0375	0.0436	0.0361	0.0054
	10%	[21]	0.1998	0.0881	0.2031	0.1373	0.1170	0.1730	0.1282	0.1087	0.1286	0.1295	0.1413	0.0383
		Ours	0.0511	0.0294	0.0451	0.0384	0.0444	0.0649	0.0446	0.0393	0.0426	0.0434	0.0443	0.0091
	20%	[21]	0.2266	0.0903	0.1889	0.1448	0.1536	0.2245	0.0976	0.1719	0.1378	0.1317	0.1568	0.0468
		Ours	0.0494	0.0309	0.0436	0.0487	0.0531	0.0707	0.0488	0.0457	0.0468	0.0472	0.0485	0.0097
	30%	[21]	0.2474	0.1562	0.1943	0.1641	0.1702	0.2441	0.1110	0.1315	0.1467	0.1250	0.1690	0.0469
		Ours	0.0469	0.0335	0.0440	0.0517	0.0558	0.0746	0.0544	0.0494	0.0509	0.0526	0.0514	0.0103
	40%	[21]	0.2696	0.1709	0.2418	0.1593	0.1849	0.2751	0.2733	0.1574	0.1609	0.1383	0.2031	0.0551
		Ours	0.0492	0.0353	0.0454	0.0539	0.0578	0.0777	0.0587	0.0524	0.0565	0.0572	0.0544	0.0108
	50%	[21]	0.2060	0.1738	0.2653	0.1709	0.1946	0.2341	0.3000	0.1617	0.1749	0.1699	0.2051	0.0468
		Ours	0.0506	0.0372	0.0469	0.0571	0.0566	0.0816	0.0624	0.0575	0.0601	0.0604	0.0570	0.0115
Outdoor	0%	[21]	0.0016	0.0030	0.0037	0.0024	0.0026	0.0019	0.0030	0.0022	0.0030	0.0036	0.0027	0.0006
		Ours	0.0014	0.0031	0.0036	0.0025	0.0028	0.0018	0.0030	0.0020	0.0029	0.0034	0.0026	0.0007
	10%	[21]	0.0060	0.0033	0.0035	0.0025	0.0031	0.0125	0.0122	0.0089	0.0140	0.0127	0.0079	0.0046
		Ours	0.0066	0.0029	0.0036	0.0025	0.0033	0.0127	0.0128	0.0096	0.0148	0.0142	0.0083	0.0050
	20%	[21]	0.0085	0.0068	0.0059	0.0072	0.0044	0.0105	0.0109	0.0113	0.0195	0.0209	0.0106	0.0055
		Ours	0.0082	0.0057	0.0056	0.0064	0.0038	0.0105	0.0111	0.0113	0.0184	0.0190	0.0100	0.0052
	30%	[21]	0.0190	0.0127	0.0093	0.0115	0.0078	0.0088	0.0080	0.0119	0.0227	0.0187	0.0130	0.0052
		Ours	0.0178	0.0112	0.0083	0.0101	0.0070	0.0086	0.0075	0.0116	0.0203	0.0179	0.0120	0.0048
	40%	[21]	0.0347	0.0251	0.0167	0.0176	0.0107	0.0075	0.0071	0.0092	0.0207	0.0164	0.0166	0.0086
		Ours	0.0354	0.0237	0.0150	0.0152	0.0096	0.0073	0.0068	0.0094	0.0186	0.0167	0.0158	0.0087
	50%	[21]	0.0448	0.0361	0.0230	0.0215	0.0137	0.0074	0.0077	0.0074	0.0182	0.0139	0.0194	0.0126
		Ours	0.0459	0.0354	0.0210	0.0195	0.0124	0.0070	0.0074	0.0075	0.0171	0.0147	0.0188	0.0127

**Table 2:** A quantitative comparison of the number of optimization iterations and the reduced cost values between our proposed algorithm and [21] on indoor (DuoDIC [24]) and outdoor (KITTI2015 [25,26]) datasets by adjusting the left camera at different angles while increasing the outlier ratio.

Scenario	Outlier Ratio	Algorithm	0∼1 deg.		2∼3 deg.		4∼5 deg.		6∼7 deg.		8∼9 deg.		Mean ↓		Std. ↓	
			# of iter.	Cost	# of iter.	Cost	# of iter.	Cost	# of iter.	Cost	# of iter.	Cost	# of iter.	Cost	# of iter.	Cost
Indoor	0∼10%	[21]	14.8	0.0008	17.1	0.0009	15.1	0.0009	14.8	0.0010	15.0	0.0010	15.3	0.0009	0.9836	$9.6885\times 10^{-5}$
		ours	16.1	0.0004	15.5	0.0005	16.1	0.0006	16.2	0.0005	16.7	0.0005	16.1	0.0005	0.4454	$5.9028\times 10^{-5}$
	20∼30%	[21]	19.1	0.0015	18.4	0.0017	13.5	0.0018	16.3	0.0019	14.6	0.0018	16.4	0.0017	2.4045	0.0001
		ours	15.5	0.0007	15.2	0.0010	15.8	0.0012	16.1	0.0011	16.3	0.0009	15.8	0.0010	0.4668	0.0001
	40∼50%	[21]	21.7	0.0017	13.5	0.0019	14.1	0.0021	17.1	0.0022	13.8	0.0021	16.1	0.0020	3.4648	0.0001
		ours	15.5	0.0008	15.4	0.0010	15.3	0.0013	16.3	0.0013	16.7	0.0011	15.8	0.0011	0.6364	0.0002
Outdoor	0∼10%	[21]	14.7	0.0030	14.3	0.0040	14.7	0.0039	14.3	0.0043	13.5	0.0046	14.3	0.0040	0.4853	0.0006
		ours	16.4	0.0023	15.0	0.0032	15.4	0.0031	16.0	0.0034	19.4	0.0036	16.5	0.0031	1.7508	0.0005
	20∼30%	[21]	12.6	0.0054	12.6	0.0071	12.8	0.0072	11.5	0.0076	11.3	0.0080	12.2	0.0071	0.6855	0.0010
		ours	14.3	0.0042	10.9	0.0057	11.2	0.0058	11.2	0.0062	12.4	0.0065	12.0	0.0057	1.4131	0.0009
	40∼50%	[21]	10.4	0.0072	12.5	0.0084	12.7	0.0079	11.8	0.0082	11.7	0.0084	11.8	0.0080	0.9115	0.0005
		ours	12.4	0.0058	11.4	0.0067	11.4	0.0062	11.8	0.0066	12.4	0.0067	11.9	0.0064	0.5184	0.0004

## Data Availability

Data are contained within the article.
